# Predicting clinical outcomes of cancer patients with a p53 deficiency gene signature

**DOI:** 10.1038/s41598-022-05243-6

**Published:** 2022-01-25

**Authors:** Evelien Schaafsma, Eric M. Takacs, Sandeep Kaur, Chao Cheng, Manabu Kurokawa

**Affiliations:** 1 Department of Biomedical Data Science, Geisel School of Medicine at Dartmouth, Hanover, NH 03755 USA; 2 Department of Biological Sciences, Kent State University, Kent, OH 44242 USA; 3 Department of Medicine, Baylor College of Medicine, Houston, TX 77030 USA

**Keywords:** Computational biology and bioinformatics, Gene regulatory networks, Biomarkers, Prognostic markers, Cancer

## Abstract

The tumor suppressor p53, encoded by the *TP53* gene, is mutated or nullified in nearly 50% of human cancers. It has long been debated whether *TP53* mutations can be utilized as a biomarker to predict clinical outcomes of cancer patients. In this study, we applied computational methods to calculate p53 deficiency scores (PDSs) that reflect the inactivation of the p53 pathway, instead of *TP53* mutation status. Compared to *TP53* mutation status, the p53 deficiency gene signature is a powerful predictor of overall survival and drug sensitivity in a variety of cancer types and treatments. Interestingly, the PDSs predicted clinical outcomes more accurately than drug sensitivity in cell lines, suggesting that tumor heterogeneity and/or tumor microenvironment may play an important role in predicting clinical outcomes using p53 deficiency gene signatures.

## Introduction

The tumor suppressor p53, also known as the guardian of the genome, is a transcription factor encoded by the *TP53* gene in humans. It acts as a sensor of cellular homeostasis and plays important roles in DNA repair, senescence, metabolism, and induction of apoptotic and non-apoptotic cell death in order to maintain the integrity of the genome and normal cellular functions. It can activate a myriad of target genes involved in cell cycle arrest, metabolism, and cell death in response to DNA damage, hypoxia, oxidative stress, and DNA mutations, resulting in the restriction of tumor development^1^. Considering its importance in key cellular processes, *TP53* is found to be mutated in ~ 50% of human cancers, more than any other gene in human cancers^2^. In this regard, Li–Fraumeni syndrome is a familial disorder that is associated with a *TP53* mutation which predisposes carriers to a broad spectrum of cancers^3^. Also, a number of knockout mouse studies showed that the loss of the *TP53* gene leads to the development of spontaneous tumors, particularly sarcoma and lymphoma^4^.

In the past decades, much effort has been focused on predicting clinical outcomes based on *TP53* status in a variety of cancers. Some studies have successfully shown that *TP53* mutations/deletion are linked to a poorer prognosis in certain cancer types, such as head and neck, liver, and hematopoietic cancers^5^. However, numerous conflicting results have been reported for cancers of the bladder, brain, breast, lung, colon, esophagus, and ovary^5^. For instance, there were approximately 1157 reports on lung and bronchus cancers showing that *TP53* mutations were associated with poor survival, while 1167 reports showed no such association^5^. Therefore, based on these studies, it appears that no simple generalization can be made regarding the association of *TP53* mutation status with clinical outcomes. Importantly, it is known that some of the p53 mutants, if not all, show gain-of-function rather than loss-of-function mutations, indicating that p53 mutants can induce genes that wild type p53 cannot control. Supporting this, recent studies suggest that the prognostic value of the *TP53* mutation may depend on the mutated residue^6,7^, cancer type, presence of mutation in other genes^8^, and clinical backgrounds^9^. Most importantly, *TP53* mutation status is not the only factor affecting the p53 signaling pathway. Even if cancer cells carry wild type p53, its activity is often suppressed upstream by negative regulators, such as MDM2 and MDM4^10,11^, or downstream by promoter methylation of p53 target genes^12,13^.

Given the complexity of the regulation of p53 and its target expression, p53 activity is expected to be a better prognostic marker than *TP53* mutation status. Our group has previously demonstrated that the p53 deficiency score (PDS), which is inferred based on expression levels of p53 regulated genes, is a better predictor of recurrence in early-stage lung adenocarcinoma patients than *TP53* mutation status^14^. In the present study, we extended the computational approach to include 20 cancer types to analyze the association between PDS and clinical outcomes.

## Results

**Figure 1:**
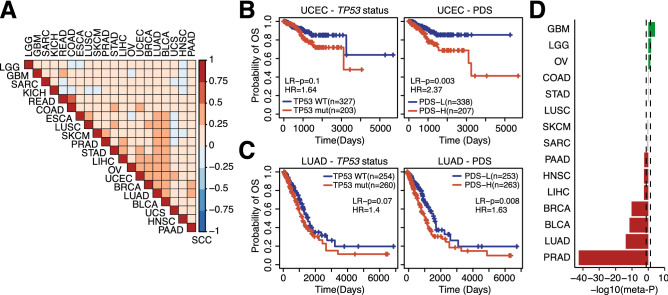
Prognostic associations in TCGA and PRECOG. (**A**) Correlation matrix of *TP53* signatures using Spearman correlation coefficients (SCC). (**B**) KM plots for UCEC in TCGA based on *TP53* mutation status (left) or p53 deficiency scores (PDS) (right). (**C**) KM plots for LUAD in TCGA based on *TP53* mutation status (left) or PDS (right). *LR* p-value calculated between groups using Log-rang tests, *HR* hazard ratio. (**D**) Meta-p-values for association between PDSs and prognosis in PRECOG. Bars indicate − log10(meta p-values). Negative values indicate a hazardous role for PDSs, while positive values represent protective associations. LGG (brain lower grade glioma), GBM (glioblastoma multiforme), SARC (sarcoma), KICH (kidney chromophobe), READ (rectum adenocarcinoma), COAD (colon adenocarcinoma), ESCA (esophageal carcinoma), LUSC (lung squamous cell carcinoma), SKCM (skin cutaneous melanoma), PRAD (prostate adenocarcinoma), STAD (stomach adenocarcinoma), LIHC (liver hepatocellular carcinoma), OV (ovarian serous cystadenocarcinoma), UCEC (uterine corpus endometrial carcinoma), BRCA (breast invasive carcinoma), LUAD (lung adenocarcinoma), BLCA (bladder urothelial carcinoma), UCS (uterine carcinosarcoma), HNSC (head and neck squamous cell carcinoma), PAAD (pancreatic adenocarcinoma).

We have previously proposed a computational method to predict sample-specific PDS in lung adenocarcinoma based on the expression of genes that are associated with *TP53* gene mutation status^14^. Here, we extended this method to 19 additional cancer types from The Cancer Genome Atlas (TCGA). These cancer types were selected based on having at least 20 patients who had protein-altering *TP53* mutations and made up at least 10% of all patients within a cancer type. The total number of patients with *TP53* mutations in these 20 cancer types was 3461, which constituted 47% of all included samples (Table 1). Cancer-specific mutation rates ranged from 93% for ovarian cancer to 12% for prostate cancer (Table 1). For each cancer type, we generated p53 deficiency signatures in which genes are weighted based on their association with *TP53* mutation status; genes highly associated with *TP53* mutations receive high weights, whereas uncorrelated genes receive small weights. The resulting weight profiles were mildly but positively correlated (Fig. 1A), with the highest correlation being between the rectum adenocarcinoma and colon adenocarcinoma signatures (Spearman Correlation Coefficient = 0.583, p < 2E−323).

**Table 1:** *TP53* status of each cancer types.

Cancers	# Samples	# *TP53* mutant	# *TP53* wild type	% Mutation
OV	206	192	14	93.2
UCS	57	52	5	91.2
ESCA	184	158	26	85.9
LUSC	482	403	79	83.6
READ	146	107	39	73.3
HNSC	501	353	148	70.5
PAAD	169	104	65	61.5
COAD	405	223	182	55.1
LUAD	512	260	252	50.8
LGG	524	257	267	49.0
STAD	411	201	210	48.9
BLCA	406	198	208	48.8
UCEC	529	203	326	38.4
SARC	237	86	151	36.3
GBM	158	55	103	34.8
BRCA	1023	349	674	34.1
KICH	66	21	45	31.8
LIHC	360	108	252	30.0
SKCM	467	72	395	15.4
PRAD	494	59	435	11.9

The percentage of surviving patients is high for most TCGA cancer types, which makes it difficult to identify prognostic associations due to low statistical power. We selected cancer types with a relatively high death event percentage (> 50%). Within these cancer types, we observed significant differences in survival, including uterine corpus endometrial carcinoma (log-rank p = 0.003) and lung adenocarcinoma (log-rank p = 0.008) (Fig. 1B,C). Interestingly, there was no statistical association between overall survival and *TP53* mutation status in these cancer types (Fig. 1B,C). In fact, *TP53* mutation status was only prognostic in pancreatic adenocarcinoma (log-rank p = 2E−3). To more comprehensively evaluate the association between PDS and patient prognosis, we also utilized the PRECOG dataset^15^, a comprehensive collection of more than 100 gene expression datasets with high-quality survival data from patients treated with standard-of-care chemotherapy. We confirmed the prognostic relevance of PDS in uterine corpus endometrial carcinoma and lung adenocarcinoma and found a number of additional cancer types in which p53 deficiency was associated with prognosis. Generally, high PDSs were associated with shorter survival; high PDSs were hazardous in patients with prostate adenocarcinoma, bladder urothelial carcinoma, breast invasive carcinoma, liver hepatocellular carcinoma, and head and neck squamous carcinoma (Fig. 1D). Interestingly, brain cancers including glioblastoma multiforme and brain lower grade glioma showed a negative association with survival, as well as ovarian serous cystadenocarcinoma (Fig. 1D). These findings suggest that under standard-of-care chemotherapy, p53 deficiency is associated with poorer survival outcomes in about half of the evaluated cancer types; exceptions were observed in brain cancers in which p53 deficiency is associated with improved survival.

**Figure 2:**
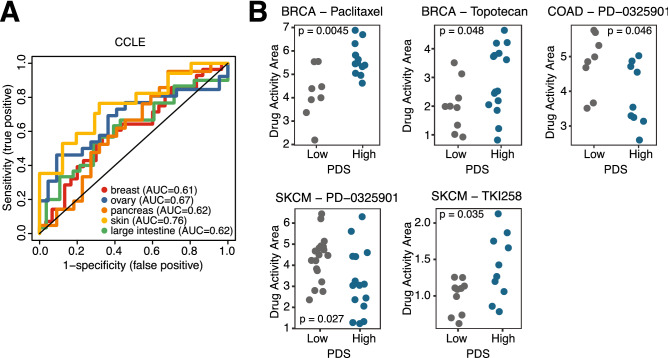
Drug sensitivity in CCLE data. (**A**) AUC curves of PDS predicting *TP53* mutation status for cancer types with AUC scores > 0.60. (**B**) Differences in drug sensitivity based on PDS status using the median of PDSs to demarcate low and high p53 deficiency groups. p-value calculated by two-sided Student’s t-test.

Next, we examined whether PDSs can predict drug sensitivity in cancer cell lines. Towards this end, we inferred PDSs in the Cancer Cell Line Encyclopedia (CCLE) dataset which contains drug sensitivity data on different drugs in a wide range of cell lines^16^. We could only confidently capture p53 deficiency in a limited number of cancer types (AUC of predicting *TP53* mutation status > 0.60) (Fig. 2A). We thus evaluated associations between PDSs and drug sensitivity in five cancer types for which we could confidently capture p53 deficiency (breast invasive carcinoma, colon adenocarcinoma, ovarian serous cystadenocarcinoma, pancreatic adenocarcinoma, and skin cutaneous melanoma). We also only included drugs for which at least 10 cell lines for the given cancer type were tested, resulting in the evaluation of 11 drugs for breast invasive carcinoma and up to 15 drugs for skin cutaneous melanoma. A small number of evaluated associations was statistically significant (Fig. 2B). In two out of five cases (colon adenocarcinoma and skin cutaneous melanoma), p53 deficiency was associated with increased drug sensitivity, whereas in the other three comparisons (breast invasive carcinoma, ovarian serous cystadenocarcinoma, and pancreatic adenocarcinoma), p53 deficiency was associated with decreased drug sensitivity (Fig. 2B).

**Figure 3:**
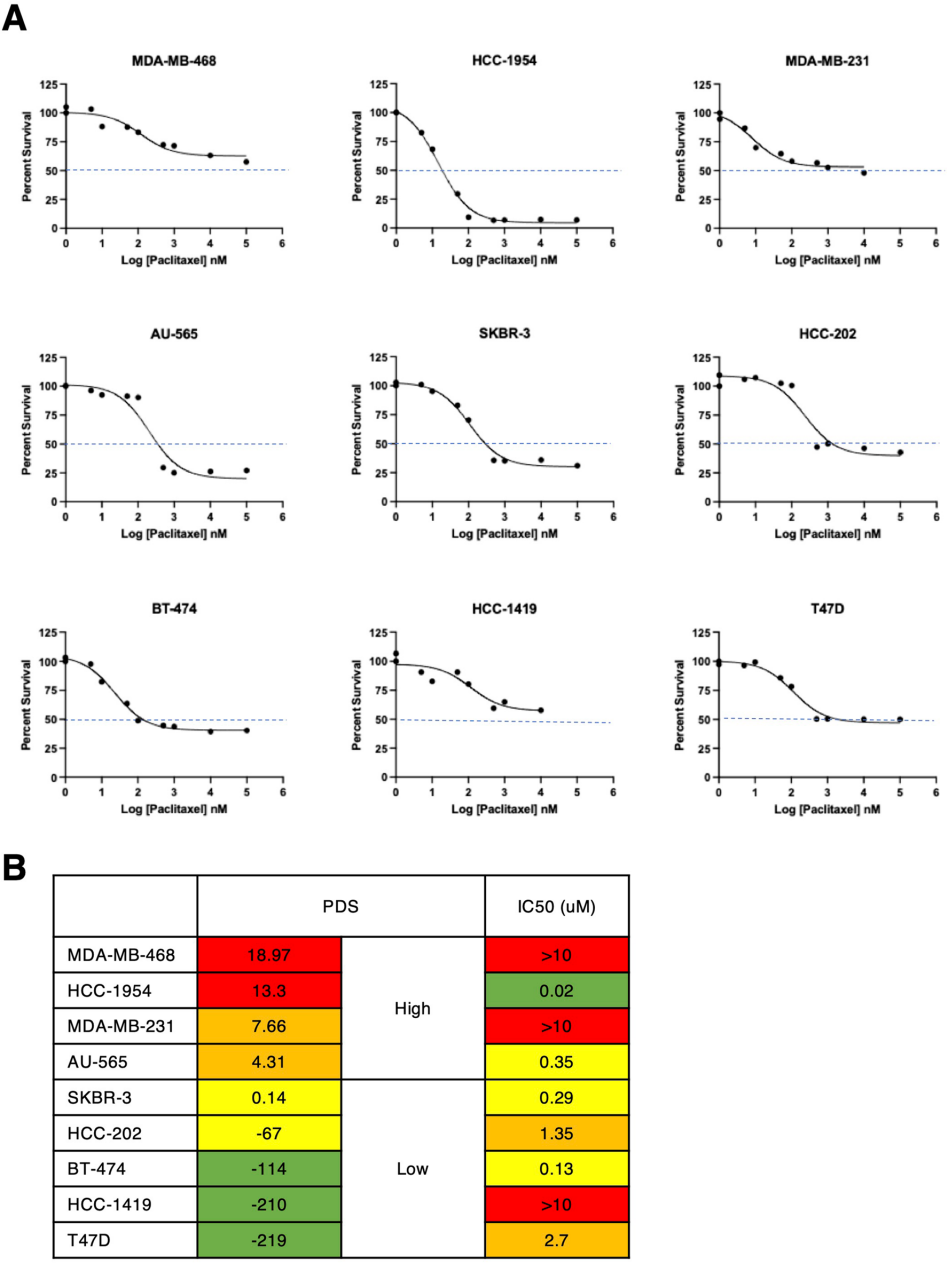
Drug sensitivity in breast cancer cell lines. (**A**) The indicated breast cancer cell lines were plated into 96-well plates and were treated with various concentrations of paclitaxel. MTS assays were performed following a 48-h incubation with paclitaxel. (**B**) PDSs and paclitaxel IC50’s are shown for each breast cancer cell line.

To further investigate the relationship between the *TP53* gene expression signature and drug sensitivity at the cell level, we analyzed p53 deficiency and paclitaxel sensitivity by an actual cell culture model of breast cancer where a strong inverse association between p53 deficiency and paclitaxel sensitivity was observed (Fig. 2B). We treated 9 breast cancer cell lines endogenously expressing a mutant p53 (MDA-MB-468, HCC-1954, MDA-MB-231, AU-565, SKBR-3, HCC-202, BT-474, HCC1419, and T47D) with various concentrations of paclitaxel in vitro. Forty-eight hours later, we performed MTS assays and calculated paclitaxel IC50’s for each cell line (Fig. 3A). Supporting the CCLE dataset analysis (Fig. 2B), the MDA-MB-468 and MDA-MB-231 cell lines, which showed high PDSs, were highly resistant to paclitaxel (Fig. 3A,B). In contrast, the HCC-1954 cell line was sensitive to paclitaxel (Fig. 3A), even though it had a high PDS (Fig. 3B). Conversely, the HCC-1419 cell line, which showed a low PDS (Fig. 3B), was resistant to paclitaxel (Fig. 3A). Overall, among the 9 cell lines tested, there was a mild association between high PDSs and paclitaxel resistance in vitro (Fig. 3B), with 2 out of 4 cell lines in the high PDS category being resistant to paclitaxel and only 1 out of 5 cell lines being resistant in the low PDS category. These results suggest that PDSs can be used as a prognostic marker for clinical samples and in some cell lines. The lower association between PDSs and treatment response in cell lines could be due to the inherent differences between gene expression profiles of tumor tissues and cell lines, including the absence of surrounding stromal cells and immune cells as well as the lack of substantial tumor cell heterogeneity in cell lines. In addition, some cell lines are equipped with mechanistic aspects that could cause drug resistance independently of p53 activity. For example, the breast cancer cell line MCF7 expresses wild type p53. However, the lack of caspase-3 makes this cell line extremely resistant to cell death stimuli, including paclitaxel, despite its low PDS (score − 203).

**Figure 4:**
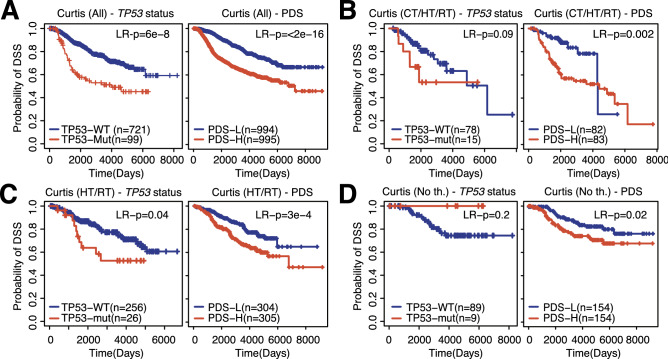
PDSs in the METABRIC/Curtis dataset. (**A**) KM plot evaluating the association of *TP53* mutation status (left) or PDS (right) with survival in the METABRIC/Curtis dataset (all patients). (**B**) Association of *TP53* mutation status (left) or PDS (right) with survival for patients receiving a combination of chemotherapy therapy, hormone therapy, and radiotherapy. (**C**) Association of *TP53* mutation status (left) or PDS (right) with survival for patients receiving a combination of hormone therapy and radiotherapy. (**D**) Association of *TP53* mutation status (left) or PDS (right) with survival for patients not receiving any treatment. *LR* p-value calculated between *TP53* groups using Log-rang tests, *Cox-p* p-value calculated using Coxph regression using PDSs as continuous variable, *DSS* disease-specific survival.

Cancer patients often undergo different treatment schemes. To evaluate the effect of different treatment modalities in tumor gene expression data, we applied the breast cancer-specific PDS to the METABRIC (Curtis) dataset^17^, which contains gene expression data from patients treated by a variety of therapies. Overall, PDSs were hazardous (Fig. 4A), consistent with our earlier findings in Fig. 1. When comparing low and high PDSs in patients treated with the same therapy, significant differences in survival were observed; p53 deficiency was always associated with poor prognosis. This association reached statistical significance for the chemotherapy/hormone therapy/radiotherapy combination (log-rank p = 0.002) (Fig. 4B), the hormone therapy/radiotherapy combination (log-rank p = 3E−4) (Fig. 4C) and for patients that did not receive any therapy (log-rank p = 0.02) (Fig. 4D). No significant relationship was observed within the patient group treated with only chemotherapy (data not shown). Consistent with our earlier findings (Fig. 1B,C), p53 deficiency was a better predictor than *TP53* mutation status (Fig. 4A–D).

**Figure 5:**
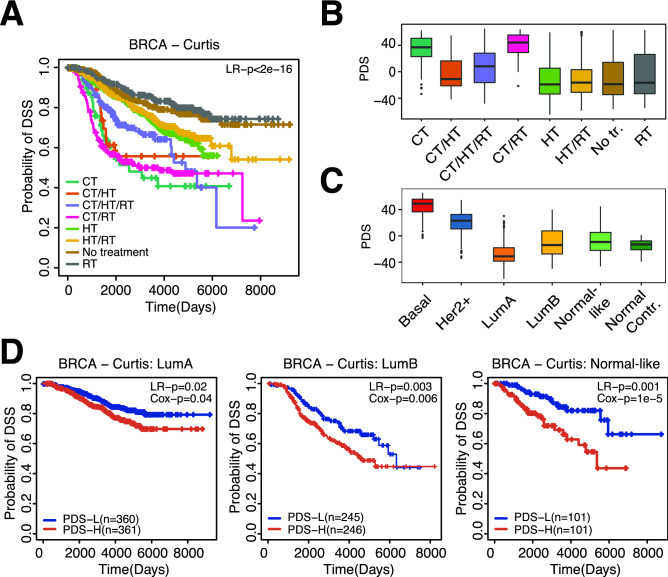
PDSs in breast cancer. (**A**) Differences in survival comparing different treatment strategies in the METABRIC/Curtis dataset. *LR-p* p-value calculated between *TP53* groups using Log-rank test, *DSS* disease-specific survival. (**B**) PDSs compared between distinct treatment groups. (**C**) PDSs for breast cancer subtypes in METABRIC/Curtis dataset. (**D**) KM plots evaluating the association between PDS and survival in the METABRIC/Curtis dataset within PAM50 breast cancer subtypes. *LR-p* p-value calculated between *TP53* groups using Log-rank test, *Cox-p* p-value calculated using Coxph regression using PDSs as continuous variable, *DSS* disease-specific survival.

The comparison of the survival probability among treatments also revealed differential survival among patient groups treated with different therapies. For example, patients receiving a combination of chemotherapy/hormone therapy/radiotherapy survived much longer compared to patients who only received chemotherapy (Fig. 5A). Interestingly, some treatment groups were associated with PDSs. For instance, the chemotherapy (CT) and chemotherapy/radiotherapy (CT/RT) groups of patients had higher PDSs as compared to other treatment groups (Fig. 5B). Since patients within this dataset were not randomized into treatment groups, it is possible that breast cancer subtypes contribute to this discrepancy as subtypes play a major role in determining treatment options. We found that basal and HER2+ breast cancer patients have much higher PDSs compared to the other subtypes (Fig. 5C) and these are the breast cancer subtypes with the poorest survival^18^. Compared to basal and HER2 + breast cancers, the Luminal A, Luminal B, and normal-like subtypes are known to be associated with better prognosis. Notably, PDSs predicted patients’ survival within these subtypes (Fig. 5D).

## Discussion

Gene expression signatures are emerging as powerful prognostic markers of clinical outcomes as compared to mutation status of a small gene set. Previously, we have generated a computational p53 deficiency gene signature model^14^. In the present study, we sought to investigate whether the PDS also predicts clinical outcomes in other cancer types. We have shown that PDS is a better predictor of clinical outcomes as compared to *TP53* mutation status. It is important to note that the *TP53* gene expression signature can be influenced by transcription factors other than p53. For instance, although p53 is considered to be the primary transcription factor for this signature, the p53 family proteins p63 and p73 are also known to induce the expression of many p53-targeted genes^19–21^. This may at least partially explain why cell lines expressing mutant p53 had a range of PDSs (Fig. 3). In addition, clinical tumor samples likely contain non-cancer cells, such as stromal cells and immune cells, which would also affect PDSs as discussed below.

Despite high survival rates in TCGA datasets, which limits the overall statistical power of prognostic analysis, we found a significant difference in overall survival in uterine corpus endometrial carcinoma and lung adenocarcinoma, where high PDSs predicted poor overall survival (Fig. 1B). Likewise, in the PRECOG dataset, high PDSs were found to be associated with poor survival in patients with prostate adenocarcinoma, lung adenocarcinoma, bladder urothelial carcinoma, breast invasive carcinoma, liver hepatocellular carcinoma, head and neck squamous carcinoma, and pancreatic adenocarcinoma (Fig. 1C). These results are in accordance with other studies in which *TP53* gene mutation/signatures were shown to be predictive of clinical outcomes in different types of cancer^18,22–26^. Surprisingly, in glioblastoma multiforme, brain lower grade glioma, and ovarian serous cystadenocarcinoma, high PDSs predicted better survival under chemotherapy (Fig. 1C). Recently, it was reported that *TP53* mutation status alone is associated with improved survival in glioblastoma^27^. Our results support this observation. It is interesting to note that the frequency of *TP53* mutations vary among these cancer types (Table 1). The functional significance of p53 and its mutation in glioblastoma, low-grade glioma, and ovarian cancer needs to be further investigated.

Our PDS model was also validated in breast cancer subtypes. The *TP53* gene mutation frequency ranges from 12% in Luminal A, 32% in luminal B, 75% in HER2-enriched breast cancers to 84% in basal-like cancers^18^. Indeed, our analysis indicated that the PDSs were high in basal-like and HER2+ breast cancer patients, while patients with the Luminal A subtype showed the lowest scores (Fig. 4C). It is worth noting that basal-like and HER2-enriched breast cancer subtypes are known to be associated with the worst survival rates^18^. Unfortunately, patients within the METABRIC/Curtis dataset were not treated with Herceptin therapy, the most common therapy for HER2+ breast cancer patients, preventing conclusions about the association between PDSs and response to Herceptin. Nevertheless, we were able to scrutinize the impact of PDSs in other therapies based on the available data. It was found that high deficiency scores predicted poor survival in patients treated with the chemotherapy/hormone therapy/radiotherapy combination (CT/HT/RT) (Fig. 4B), the hormone therapy/radiotherapy combination (HT/RT) (Fig. 4C), and for patients who did not receive any therapy (Fig. 4D).

While PDSs were associated with treatment response in breast cancer, we noted that the relationship between p53 deficiency and treatment sensitivity was complex. A recent comprehensive analysis revealed that although mutant p53 can impact clinical outcomes negatively, wild type p53 could also mediate poor clinical responses depending on the treatments and breast cancer subtype^28^. It has been reported that a 39-gene p53 signature is predictive of poor prognosis after adjuvant tamoxifen therapy in ER+, but not ER-, breast cancer^29^. In a neoadjuvant chemotherapy setting however, *TP53* mutation status is associated with better patient outcomes in early-stage breast cancer^30–33^. In basal-like breast cancer, p53 deficiency predicts a better response to an epirubicin-cyclophosphamide regimen^34^. Thus, additional variables related to p53 deficiency likely determine treatment sensitivity. One suggested cause is that certain therapies, including DNA-damaging reagents, induce senescence in p53-proficient tumors, which may, in turn, lead to the emergence of the persister cells that secrete senescence-associated cytokines and, thereby, promote tumor cell survival^35,36^. In this regard, it is interesting to note that PDSs predicted clinical outcomes more accurately than drug sensitivity at the cell line level (Fig. 3). This could be partially ascribed to cell lines’ intrinsic resistance to cell death that operates independently of the p53 pathway. Another possibility may be explained by the fact that cell lines are homogenous clonal cells, whereas tumors are comprised of heterogeneous cell populations. While the functional significance of the loss of p53 functions in tumor cells has been extensively studied, recent studies have also shown the tumor suppressive role of p53 in stroma cells and tumor-infiltrating immune cells^37–42^. It remains to be determined to what degree the actual tumor cells, tumor-associating stroma cells, and tumor-infiltrating immune cells contribute to the *TP53* signature.

In conclusion, our study shows that PDSs are a reliable prognostic predictor of clinical outcomes in the majority of cancer types and treatments. Moreover, PDSs predict clinical outcomes better than the analysis of *TP53* mutation status. In combination with another gene signature or mutation status, the *TP53* signature may be used to predict responses to certain therapies more accurately and may help to select a better therapeutic option in certain cancers.

## Methods

All data presented in this paper are publicly available at http://firebrowse.org (TCGA), https://precog.stanford.edu (PRECOG), https://portals.broadinstitute.org/ (CCLE), or Curtis et al.^17^.

### *TCGA*

Level 3 RNA-seq data from The Cancer Genome Atlas (TCGA) project were downloaded from FireBrowse (http://firebrowse.org). RSEM-normalized gene expression data for 20,501 genes was provided^43^. Only cancer types containing at least 20 patients with protein-altering *TP53* mutations and at least a 10% protein-altering *TP53* mutation rate were included, resulting in the inclusion of 20 cancer types. These included KICH, kidney chromophobe; LGG, brain lower grade glioma; GBM, glioblastoma multiforme; BRCA, breast invasive carcinoma; LUSC, lung squamous cell carcinoma; LUAD, lung adenocarcinoma; READ, rectum adenocarcinoma; COAD, colon adenocarcinoma; UCS, uterine carcinosarcoma; UCEC, uterine corpus endometrial carcinoma; OV, ovarian serous cystadenocarcinoma; HNSC, head and neck squamous carcinoma; PRAD, prostate adenocarcinoma; STAD, stomach adenocarcinoma; SKCM, skin cutaneous melanoma; BLCA, bladder urothelial carcinoma; LIHC, liver hepatocellular carcinoma; SARC, sarcoma; PAAD, pancreatic adenocarcinoma; and ESCA, esophageal carcinoma (Table 1). TCGA somatic mutation information and clinical data were also downloaded from FireBrowse (http://firebrowse.org).

### *PRECOG*

Gene expression datasets were obtained from PREdiction of Clinical Outcomes from Genomic profiles (PRECOG, https://precog.stanford.edu)^15^ as normalized datafiles. Clinical data including overall survival times and outcomes for studies were obtained from the same resource. Datasets were filtered by tissue type to match TCGA tumor types, and only datasets with more than 40 samples and over 20% mortality rate were included in our analysis. This resulted in the inclusion of 126 datasets.

### *METABRIC/Curtis data*

Breast cancer data for the METABRIC/Curtis dataset was downloaded from a previous publication^17^. Disease-specific survival and treatment information was obtained from the same publication.

### *CCLE*

The Cancer Cell Line Encyclopedia (CCLE) data was downloaded from the CCLE database (https://portals.broadinstitute.org/)^16^. This dataset provides gene expression profiles measured by microarray experiments for more than 1100 cancer cell lines and their sensitivity to 24 anticancer drugs. Drug Activity Area was utilized as assessment of drug efficacy, where higher Drug Activity Areas indicate higher sensitivity.

### Generation of PDS signatures

For a detailed explanation of PDS signature generation, see Zhao et al.^14^. Briefly, for each cancer type, patients were separated into two groups based on the presence or absence of protein-altering *TP53* mutations. A logistic regression model was fitted for each gene using expression as the independent variable and *TP53* mutation status (0 = *TP53* wild type, 1 = *TP53* mutation) as the dependent variable. Based on the resultant p-values and β-coefficients, genes were separated into an up profile (β  > 0) and a down (β < 0) profile. For both up and down profiles, p-values were − log10 transformed, trimmed at 10 to reduce outliers, and scaled into [0, 1] to obtain the weights for genes. The resulting up and down profiles constituted the p53 deficiency signature for a cancer type.

### Calculation of PDSs

For a detailed explanation of p53 deficiency inference, see Zhao et al.^14^. Briefly, the enrichment of cancer type-specific p53 deficiency signatures in patient samples was determined by BASE^44^, a rank-based gene set enrichment method, for each utilized gene expression dataset. BASE calculated sample-specific scores, named PDSs based on their gene expression profiles measured by microarray or RNA-seq analysis. The deficiency scores indicated the degree of p53 pathway deficiency, with a higher score indicating lower p53 activity.

### Survival analysis

Survival analyses were performed using the R survival package (version 3.2-7). Log-rank tests were performed to evaluate overall or disease-specific survival probabilities between two groups using the ‘survdiff’ function. Univariate Coxph regression was performed on continuous PDSs using the ‘coxph’ function. Kaplan–Meier plots were generated using the ‘survfit’ function. For KM plots using the TCGA data, the percentage of *TP53* mutated samples was used as a cutoff to determine low and high PDSs. This was done to allow for a direct comparison between the prognostic significance of *TP53* mutations and PDSs without the confounding of different group sizes. For all other KM plots, the median PDS was used to separate low and high PDS groups.

For the survival analysis in the PRECOG dataset, PDSs were inferred in a cancer type-specific manner. After computing the PDSs, we fitted a univariate Cox regression model to measure the association between PDSs and all-cause or disease-specific mortality (if available). Z-scores were extracted from the fitted models and a meta z-score was calculated for PDSs across microarray datasets of the same cancer type. A meta z-score was calculated using the weighted Stouffer’s z-score method which uses the dataset sample size as weights. Meta p-values were calculated from the meta z-scores by referring to the standard normal distribution.

### Cell culture

The HCC-1954, HCC-202, AU-565, SKBR-3, BT-474, and T47D cell lines (ATCC) were maintained in RPMI media (Corning) supplemented with 10% fetal bovine serum (FBS) and 1% penicillin–streptomycin (Corning), whereas MDA-MB-468 and HCC-1419 (ATCC) were cultured in RPMI with 10% FBS, 1% penicillin–streptomycin (HyClone), 1% MEM non-essential amino acids (Gibco), and 100 μM sodium pyruvate (Gibco). The MDA-MB-231 cell line (ATCC) was cultured in DMEM (Corning) with 10% FBS and 1% penicillin–streptomycin (Corning). MTS assays were performed using the CellTiter 96 Aqueous One Solution Cell Proliferation Assay kit (Promega) as previously described^45^. In brief, 10,000 cells/well were plated into a 96 well plate. Twenty-four hours later, the cells were treated with paclitaxel (LC Laboratories) for an additional 48 h. DMSO and the apoptosis-inducing pan-kinase inhibitor staurosporine (1 μM) were used as negative and positive controls, respectively. The absorbance at 490 nm (OD 490 nm) was normalized against DMSO.

### Statistical analysis

The Spearman correlation coefficient was reported for correlation analyses and was calculated using the R ‘cor’ function. Area Under the Curve (AUC) values evaluating the ability of PDSs in predicting *TP53* mutation status in the CCLE dataset were calculated and plotted using the ‘pROC’ R package. The statistical difference between Drug Activity Areas between *TP53* deficiency groups in the CCLE dataset was calculated by two-sided Student’s t-tests.
